# Synthesis and characterization of magnesium ferrite-activated carbon composites derived from orange peels for enhanced supercapacitor performance

**DOI:** 10.1038/s41598-024-54942-9

**Published:** 2024-04-08

**Authors:** Reda.S. Salama, Mostafa S. Gouda, Mohamed F. Aly Aboud, Fares T. Alshorifi, A. A. El-Hallag, Ahmad K. Badawi

**Affiliations:** 1 Basic Science Department, Faculty of Engineering, Delta University for Science and Technology, Gamasa, Egypt; 2 Department of Mechanical Engineering, Faculty of Engineering, Islamic University of Madinah, P.O.B. 170, 42351 Madinah, Saudi Arabia; 3 Department of Chemistry, Faculty of Science, University of Saba Region, Marib, Yemen; 4 Department of Chemistry, Faculty of Science, Sana’a University, Sana’a, Yemen; 5 Civil Engineering Department, El-Madina Higher Institute for Engineering and Technology, Giza, 12588 Egypt

**Keywords:** Magnesium ferrites nanoparticles, Activated carbon, Agricultural wastes, Orange peel, Energy storage, Supercapacitors, Green chemistry, Materials chemistry, Energy

## Abstract

Supercapacitors have emerged as highly efficient energy storage devices, relying on electrochemical processes. The performance of these devices can be influenced by several factors, with key considerations including the selection of electrode materials and the type of electrolyte utilized. Transition metal oxide electrodes are commonly used in supercapacitors, as they greatly influence the electrochemical performance of these devices. Nonetheless, ferrites' low energy density poses a limitation. Hence, it is crucial to create electrode materials featuring unique and distinct structures, while also exploring the ideal electrolyte types, to enhance the electrochemical performance of supercapacitors incorporating magnesium ferrites (MF). In this study, we effectively prepared magnesium ferrites (MgFe_2_O_4_) supported on activated carbon (AC) derived from orange peels (OP) using a simple hydrothermal method. The resulting blends underwent comprehensive characterization employing various methods, including FTIR, XRD, TEM, SEM, EDX, and mapping analysis. Moreover, the electrochemical performance of MgFe_2_O_4_@AC composites was evaluated using GCD and CV techniques. Remarkably, the MF45-AC electrode material showed exceptional electrochemical behavior, demonstrating a specific capacitance of 870 F·g^−1^ within current density of 1.0 A g^−1^ and potential windows spanning from 0 to 0.5 V. Additionally, the prepared electrodes displayed exceptional cycling stability, with AC, MF, and MF45-AC retaining 89.6%, 94.2%, and 95.1% of their initial specific capacitance, respectively, even after 5000 cycles. These findings underscore the potential of MF-AC composites as superior electrode materials for supercapacitors. The development of such composites, combined with tailored electrolyte concentrations, holds significant promise for advancing the electrochemical performance and energy density of supercapacitor devices.

## Introduction

Throughout history, the conversion and storage of energy have held a prominent place in human civilization's development. In the contemporary era, iPads, mobile phones, and personal computers are electronic devices relying on energy storage technologies, such as batteries, that enhance our daily lives and enable efficient access to information and communication^1,2^. Electrochemical capacitors, or supercapacitors (SCs), offer tremendous potential for energy storage due to their high efficiency, high power densities, extended cycle lives, environmental friendliness, and safety^3,4^. These devices have proven useful in various applications, including providing burst-power generation for electronic devices, initiating power for fuel cells, acting as backup power sources, and contributing to hybrid power systems^5,6^. Nevertheless, their potential as energy storage solutions is somewhat constrained by their energy densities. Consequently, the development of electrode composites characterized by high working voltages, large capacitances, and extended cycling lives is essential for advancing supercapacitors toward higher energy densities^7,8^. These devices rely heavily on specific materials for their energy storage, with commonly used options including conducting polymers, carbon materials, and metal oxides^9,10^. Carbon materials are used in electrical double-layer capacitors (EDLCs), that only store charges physically. Metal oxides and conducting polymers, on the other hand, are utilized in pseudo capacitors, which store charges through electrochemical reactions. Pseudo-capacitors have higher specific capacitances and energy densities than EDLCs. Graphene, graphene oxides^11,12^, carbon nanofibers, carbon nanotubes and activated carbon^13^, are commonly used as electrode materials in EDLCs^14,15^. However, despite possessing a high reversibility, stability, excellent rate capability, and high specific surface area, supercapacitors exhibit low capacitance^16–18^. In contrast, transition metal oxides and conducting polymers like NiO^19–21^, ZnO^22^, MnO_2_^23^, and Co_3_O_4_^24^, are the primary electrode materials in pseudo-capacitor^25,26^. Although transition metal oxides showed high capacitance, they are expensive, rare, and have poor rate capability. By combining these two types of materials, however, we can obtain good rate capability higher capacitance, and stability^27^.

In recent years, AC–based materials derived from natural sources have become a popular choice for supercapacitor electrodes. These materials offer various benefits, including ease of synthesis, abundance in nature, low cost, and stability^28^. Agricultural by-products such as coconut shells, wood chips, orange peels, and bamboo residues serve as abundant sources for producing activated carbon^29–33^. The activation process involves heating the raw material in the presence of a gas, typically steam or carbon dioxide, to create a porous structure. The resulting activated carbon exhibits high surface area, numerous micro and mesopores, and excellent adsorption capabilities^34^. These characteristics render it an ideal material for use in water treatment^35^, air purification, and energy storage devices like supercapacitors^36,37^. Additionally, the utilization of agricultural wastes for activated carbon production contributes to waste reduction, providing an environmentally sustainable solution with economic benefits^38^. The electron transfer process in activated carbons during supercapacitor operation involves the adsorption and desorption of ions at the electrode surface. The nanoporous structure of AC offers a substantial surface area, facilitating the adsorption of ions, facilitating rapid charge and discharge cycles. As voltage is applied, ions are attracted to the porous surface of the activated carbon, leading to the storage of electrical energy in the form of charge. Subsequently, during discharge, the stored ions are released, resulting in the flow of electrical current. The efficient electron transfer in activated carbons contributes to the high performance and quick energy release essential for supercapacitor applications. Moreover, the sustainable nature of activated carbon derived from agricultural wastes aligns with the growing demand for eco-friendly energy storage solutions. As the world explores cleaner and more sustainable energy alternatives, the use of activated carbon from natural resources in energy storage devices continues to demonstrate its potential in contributing to a greener and more efficient energy landscape^39^. In recent studies, various hybrid materials consisting of activated carbon and ceramic oxides have been developed for supercapacitors^40^.

The MgFe_2_O_4_ composites is ceramic oxide a with a crystalline system that is face-centered cubic^41^. In spinel structures, the typical configuration involves divalent ions enclosed by oxygens, forming tetrahedral sites, and trivalent ions surrounded by oxygens, creating octahedral sites^42^. However, in the case of MgFe_2_O_4_, this structural arrangement is not strictly adhered to, primarily due to the synthesis route or conditions, leading to a partially inverse spinel. In this arrangement, trivalent metal ions partially occupy tetrahedral sites, and divalent ions partially occupy octahedral sites, thereby influencing the physicochemical properties of the ferrite^43,44^. The covalent nature of the magnesium-oxygen bond results in low mobility, whereas iron exhibits higher mobility within the MgFe_2_O_4_ structure^45^. The diverse applications of ferrites nanoparticles, particularly the MgFe_2_O_4_ spinel, due to their unique magnetic properties that offer various potential applications such as cancer therapy^46^, biosensors^47^, supercapacitors^48^ and water filtration^49^. MgFe_2_O_4_, due to its unique spinel structure and electrical properties, presents an intriguing candidate for supercapacitor applications. The ceramic nature of MgFe_2_O_4_ contributes to its stability, while the partially inverse spinel structure enhances its electrochemical properties. The utilization of MgFe_2_O_4_ as a supercapacitor material is driven by its potential to offer a combination of high capacitance, stability, and unique magnetic properties, making it a promising candidate for advancing the performance of supercapacitor devices. Atiq ur Rehman et al.^50^ have reported the use of silver spinel ferrite (AgFe_2_O_4_) and its composites AgFe_2_O_4_@ZnO for supercapacitor applications. They used AgFe_2_O_4_@10%ZnO composite to achieve a specific capacitance of 585.9 F/g with a power density of 720 W/kg and an energy density of 28 Wh/kg. Ala Manohar et al.^51^ have proposed the supercapacitor application of a composite of Ni_0.35_Mg_0.65_Fe_2_O_4_ to attain a specific capacitance of 119.04 F g^-1^ at a current density of 1 A g^-1^. Arun Kumar et al.^52^ have reported the use of transition-metal-substituted manganese ferrites composites for supercapacitor applications. They used nanocomposite of Mn_0.95_Zn_0.05_Fe_2_O_4_ composite to achieve a specific capacitance of 829 F/g. Gyan Singh et al.^53^ have proposed the supercapacitor application of Nickel ferrite doped polyaniline composites to attain a specific capacitance of 758 F g^-1^ at molar ratio of 1:1 for PANI and Nickel ferrite electrode. Improving the specific surface area, conductivity, and ion transport of ferrites using predictable synthetic methods has proven to be challenging. Overcoming these challenges involves combining the advantages of other highly conductive materials, such as carbon-based materials, with ferrites to generate composites characterized by outstanding conductivity and a large surface area.

In this study, we present a novel approach to address the challenges in supercapacitor technology by synthesizing magnesium ferrites (MgFe_2_O_4_) supported on activated carbon (AC) derived from orange peels (OP) through a straightforward hydrothermal method. This innovative synthesis not only offers an efficient and scalable route for electrode material production but also aligns with environmental sustainability, utilizing agricultural waste as a renewable resource. The choice of orange peels as the source material is motivated by their abundance and status as a readily available bio-waste resource. Our research involves a process that utilizes KOH chemical activation, leading to the formation of a 3D interconnected porous structure in the carbon material. The resulting MgFe_2_O_4_@AC composites underwent a meticulous characterization employing various techniques, including FTIR, XRD, TEM, SEM, EDX, and mapping analysis, providing a comprehensive understanding of their structural and morphological features. Crucially, the electrochemical performance of these composites was systematically evaluated through galvanostatic charge–discharge (GCD) and cyclic voltammetry (CV) techniques.

## Materials and methods

### Materials

Hydrated ferric (III) chloride (FeCl_3_·6H_2_O, > 98.0%), hydrated magnesium nitrate (Mg(NO_3_)_2_·6H_2_O, > 99.0%), sodium hydroxide (NaOH, analytical grade), hydrochloric acid (HCL, 37%, analytical grade), and potassium hydroxide (KOH, analytical grade) were obtained from Merck. Polyvinylidene fluoride (PVDF), N-Methyl-2-pyrrolidone (NMP), and carbon black were sourced from Alfa Aesar. Orange peels (OP) were collected from the farm in Damietta Governorate, Egypt, between October and December.

### Synthesis of the activated carbon

Orange peels were performed as a biowaste precursor for the formulation of AC as a modified version of a previouslyy ppublished method^28,35^. The orange peels were initially cleaned by rinsing them with distilled water to remove any impurities and dusts. The peels were subjected to drying at 120 °C for 12 h. Subsequently, the resulting dried peels were mixed in stoichiometric ratio of 1:2 with potassium hydroxide at room temperature for an hour. Then, the resultant suspension was evacuated at 80 °C, and the resultant solid was subsequently dried overnight at 110 °C. The resultant powder was carbonized in a porcelain crucible under N_2_ flow for an hour at 600 °C. The generated activated carbon (AC) was iteratively neutralized using hydrochloric acid (1.0 M) and deionized water until the pH of the filtrate reached 7. Subsequently, it was dried overnight at 120 °C. The stoichiometric molar ratio of potassium hydroxide to orange peels (2:1) and the pH (7) of the solution during stirring were carefully controlled during the process.

### Synthesis of the magnesium ferrites and magnesium ferrites @ AC

To synthesize magnesium ferrite powder, Mg(NO_3_)_2_·6H_2_O (0.512 g, 2 mmol) and FeCl_3_·6H_2_O (1.08 g, 4 mmol) were dissolved in distilled water and vigorously stirred for 1 h at a temperature of 60 °C. After 2 h, sodium hydroxide was poured drop by drop till the pH of Mixture reached to 10. The resulting precipitate was transferred to a hydrothermal Teflon-lined autoclave and maintained at 150 °C for 8 h. Following this, it underwent thorough washing and filtration with ethanol and distilled water. Subsequently, the dried powder was subjected to calcination at 550 °C for 3 h in an open-air atmosphere. On the other hand, to prepare various weight contents of magnesium ferrites supported on activated carbon was prepared by the same method using 2 g of AC that was added during the vigorous stirring process. The resulting samples were labeled as AC, MF, MF25-AC, MF45-AC, and MF65-AC for activated carbon, magnesium ferrites, and 25, 45, and 65 wt.% of magnesium ferrites supported on activated carbon, respectively.

### Characterization techniques

Various analytical techniques were employed to characterize genuine and modified activated carbon. FT-IR analysis was performed to detect the different functional groups using the MATTSON FT-IR-5000S spectrophotometer. The crystallinity and crystallite size of the prepared composites were assessed using a Philips X-ray diffractometer. The Scherrer equation was employed to determine the crystallite sizes (D) of the as-synthesized nanoparticles, as indicated in Eq. (1).

1 $$D= \frac{K \lambda }{\beta {\text{cos}}\theta }$$

where K, λ, and β are constants related to crystallite shape, X-ray wavelength, and width of the diffraction peak profile at half maximum height, respectively. As well as, N_2_ sorption measurement was conducted at –196 °C to verify surface area and pore diameter of the prepared composites utilizing a BELSORP-mini II instrument. Scanning and transmission electron microscope were applied to reveal the morphology and average particle size through Jeol JSM-6510 and JEOL-JEM 2100 F, respectively. Also, their chemical composition was measured using EDX analysis.

### Electrochemical measurements

A three-electrode configuration system was utilized to analyze the performance and stability of the materials. The electrolyte used for conducting cyclic voltammetry (CV) and galvanic charge–discharge measurements (GCD) was a 2.0 M solution of sodium sulfate (Na_2_SO_4_) in water. The working electrode slurry consisted of acetylene black, PVDF, and active materials in proportions of 80.0%, 10.0%, and 10.0% by weight, respectively^54^. This slurry was applied onto a graphite sheet measuring 1.0 cm^2^ in area, and subsequently dried at 80°C in an oven. In the electrochemical experiments, the reference electrode is a standard Ag–AgCl electrode and the counter electrode utilized is a platinum wire. All measurements, including CV and GCD, were conducted at room temperature using Metrohm Auto Lab Potentiostat/Galvanostat instruments. During CV, the scan rates varied from 5.0 to 50.0 mV s^-1^. While, GCD was performed in 2.0 M aqueous Na_2_SO_4_, with specific current density ranging from 1.0 to 20.0 mA cm^-2^. To calculate the capacitance of the prepared electrodes, the galvanostatic charge–discharge equation (Eq. 2) was employed^55^:

2 $${C}_{s}(F/g)=\frac{\mathrm{I }.\mathrm{ t}}{\mathrm{m }.\mathrm{ \Delta V}}$$

where t (sec), I (mA), V (V) and m (g) are the discharge time, discharge current, V is a potential window range, and m is the active material mass loading on a working electrode.

The dual-electrode system configuration, as earlier outlined by Toyoko Imae et al.^56^, involves connecting the reference and counter electrode to one side of the cell, while the working-electrode sensing and power are connected from the other side of the electrochemical workstation. In this system, two composite electrodes, which may have similar or different compositions, are enclosed in filter paper saturated with an electrolyte The filter paper plays a dual role in this setup. Firstly, it absorbs and holds the electrolyte, ensuring its presence within the system. Secondly, it acts as a barrier, separating the two composite electrodes from each other. The created electrodes underwent CV and GCD analysis to determine energy density, power density, and specific capacitance from the following Equations^57,58^:

3 $$E (W h/Kg)=\frac{\mathrm{Cs }. {{\text{V}}}^{2}}{2}* \frac{1000}{3600}$$

4 $$P \left(W/Kg\right)=\frac{{\text{E}}}{{\text{t}}}*3600$$

## Results and discussion

### FTIR Spectra

**Figure 1:**
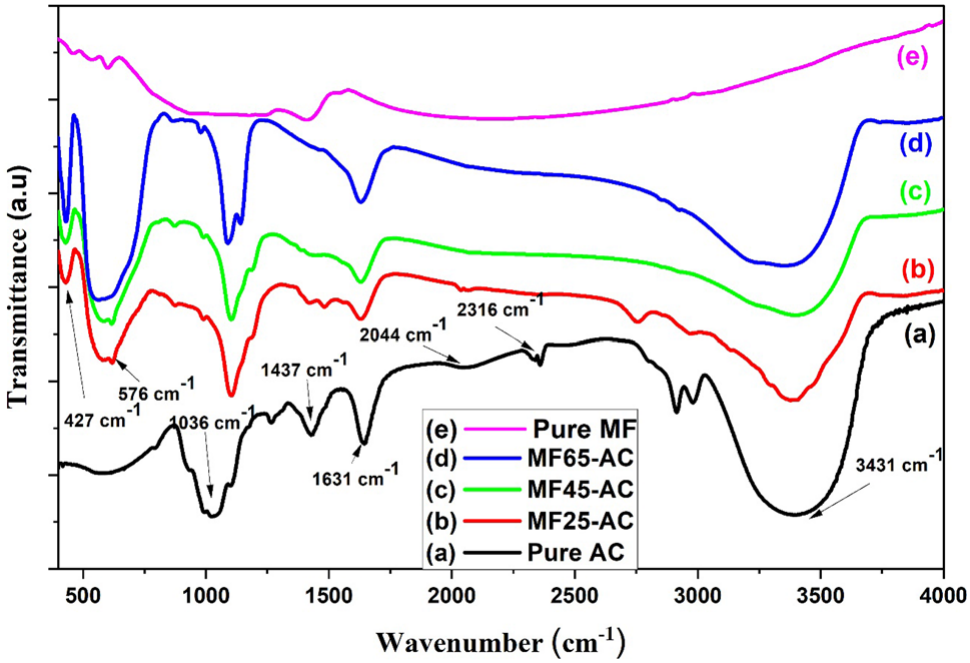
FTIR spectra of (**a**) pure AC, (**b**) MF25-AC, (**c**) MF45-AC, (**d**) MF65-AC and (**e**) Pure MF.

FTIR Spectra of the composites were displayed in Fig. 1. Figure 1a displayed that all the bands attribute to the structure of pure activated carbon. A broad band were displayed at ca. 3398 cm^-1^ that associated to stretching vibrations of hydroxyl groups (OH) in adsorbed water molecules either on the surface or within the pores of activated carbon (AC). As well as, the weak peaks around 3000 and 2950 cm^−1^ in the IR spectrum can be attributed to C–H stretching vibrations. Specifically, the peak around 3000 cm^−1^ corresponds to the stretching vibrations of C–H bonds in aliphatic hydrocarbons, while the peak near 2950 cm^−1^ is associated with C–H stretching in methyl (CH_3_) and methylene (CH_2_) groups. The presence of these peaks is indicative of the organic nature of the precursor material used in the synthesis of activated carbon and its derivatives. These C–H stretching vibrations are commonly observed in biomass-derived carbonaceous materials, such as activated carbon derived from natural sources^59,60^. Moreover, the existence of a peak observed at 1631 cm^-1^ may be associated with the bending vibrations of OH in adsorbed water molecules or the stretching of C=O in carboxylic acids^61–63^. Additional peaks observed at 2316 and 2044 cm^-1^ are assigned to the stretching vibrations of C=C and C=N, respectively^64^. While the peak at 1437 cm^-1^ was related to C–H bending vibrations, both symmetric and asymmetric^65^. Another sharp peak was also seen at 1036 cm^-1^, that was assigned to the stretching vibrations of the C–H out-of-plane^66^. Alternatively, FTIR spectra of magnesium ferrite supported on AC were demonstrated in Fig. 1(b–d). The figure two major bands at wavenumber centered at 576 and 427 cm^−1^. The wavenumbers associated with MgFe2O4 were assigned to two frequency bands, ν1 and ν2, which corresponded to the tetrahedral and octahedral sites, respectively, within the spinel structure^67^. The intensity of both bands increased as the ferrite content increased, reaching its maximum value at MF65-AC.

### X-ray diffraction pattern (XRD)

**Figure 2:**
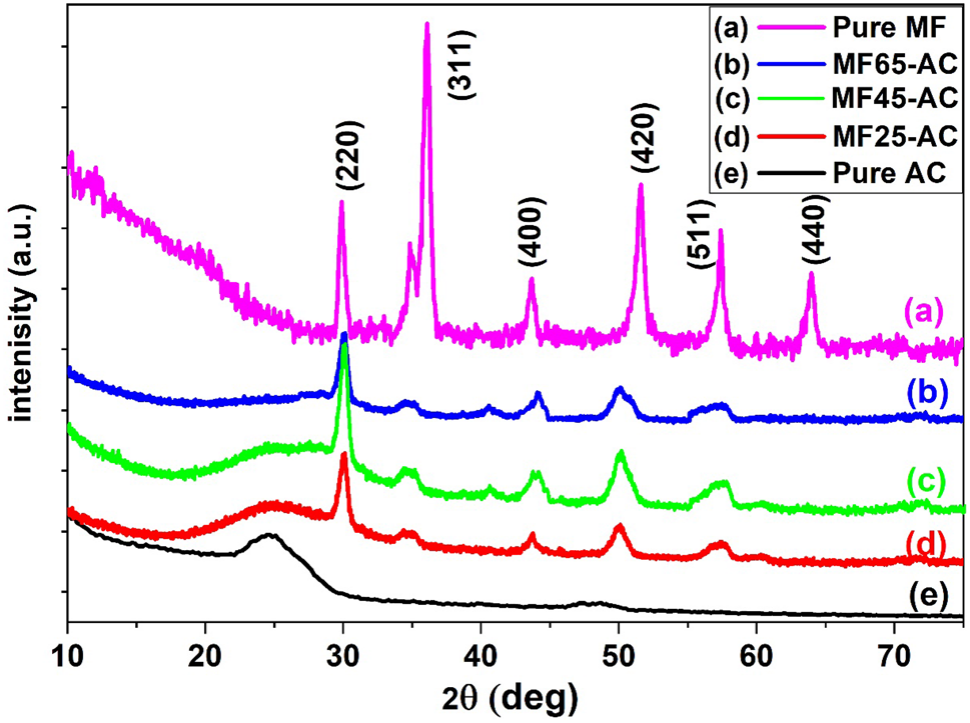
XRD patterns of pure and modified magnesium ferrites supported on activated carbon composites.

XRD patterns were performed on unmodified and modified activated carbon as shown in Fig. 2. XRD patterns of activated carbon (Fig. 2e) typically reveals two broad peaks centered around 2θ equal to 25° and 48°, which is indicative of its amorphous nature^32^. As well as, Fig. 2(a–d) showed the XRD patterns of pure and modified magnesium ferrites supported on AC. The figure displayed that sharp Bragg peaks at 2Ɵ equal 30.47°, 36.97°, 37.08°, 43.09°, 53.09°, 55.78°, and 63.05°, that are related to crystal planes of cubic spinel structure (220), (311), (400), (420), (511), and (440), respectively^68^. The intensity of the peaks was enlarged with a growth in the contents of magnesium ferrites, which reveals crystallinity improvement. To determine the lattice constant, one can employ methods like X-ray diffraction analysis, with subsequent application of Bragg's Law (nλ = 2d_220_sinθ) to interpret diffraction patterns. In our study, the lattice constants (a_o_ = 2d_220_/√3) were calculated for various samples: MF, MF25-AC, MF45-AC, and MF65-AC. The resulting values were found to be 5.5, 4.9, 5.2, and 5.4 Å, respectively, showcasing subtle variations in the lattice constants among these materials. These values signify the interatomic spacing within the crystal lattice, providing insights into the structural characteristics of each sample. As well as, the crystallite sizes of unsupported MF, MF25-AC, MF45-AC and MF65-AC were calculated from Scherrer equations and were found to be 15.74, 7.14, 7.61 and 11.47 nm, respectively. The main reason for decreasing the value of crystallite size in supported samples contrasted to pure MF could be attributed to the role of ACs in the dispersion of ferrites nanoparticles on its their surface. Also, the crystallite size increased with growing the content of magnesium ferrites because of the accumulation of MF NPs on AC surface until reached a maximum value at MF65-AC. On the other hand, the dislocation density and lattice strain can be calculated from Eqs. (5) and (6) ^69,70^and displayed in Table 1.

**Table 1:** surface and textural properties of pure AC, pure MF, MF25-AC, MF45-Ac and MF65-AC.

Sample name	Lattice constant (a_o_, Å)	Crystallite size (nm) by XRD	Lattice strain (10^–3^)	Dislocation density (δ) ×10^15^	S_BET_ (m^2^/g)	Pore diameter (Å)	Crystallite size (nm) by TEM	Specific capacitance (F/g) at 1 A/G
Pure AC	–	–	–	–	2134	18.7	–	96
Pure MF	5.5	15.47	25.7	4.18	178	6.8	16.14	402
MF25-AC	4.9	7.14	57.9	19.61	1897	17.4	8.35	516
MF45-AC	5.2	7.61	53.1	17.27	1645	16.1	9.64	870
MF65-AC	5.4	11.47	34.9	7.61	1223	13.5	12.1	800

5 $$\mathrm{dislocation\, density }\left(\updelta \right)=\left(\frac{1}{{D}^{2}}\right)$$

6 $$\mathrm{lattice\, strain }\left(\mathrm{\alpha }\right)=\left(\frac{{2\pi }^{2}}{45* ({3{\text{tan}}\theta )}^{1/2}}\right) \beta$$

### Nitrogen sorption measurements

**Figure 3:**
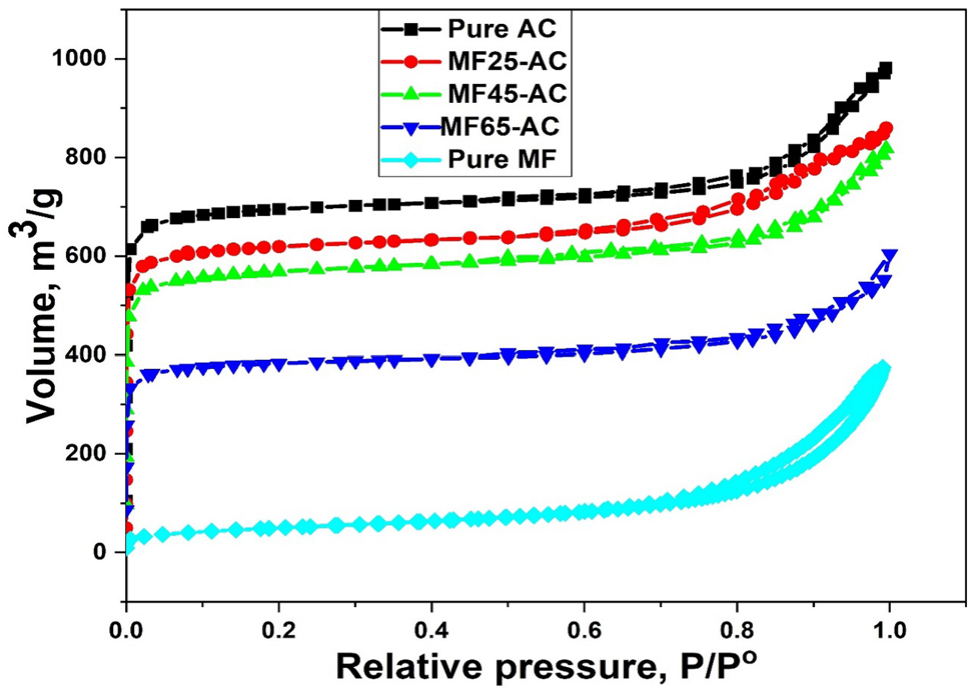
N_2_ sorption isotherms of AC, MF, MF25-AC, MF45-AC and MF65-AC.

**Figure 4:**
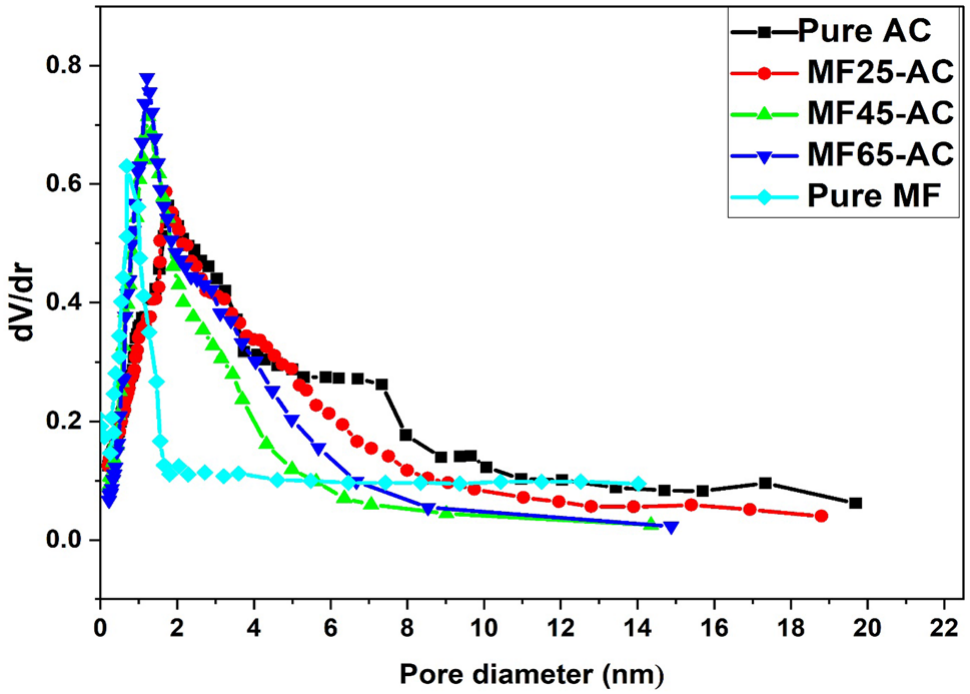
curves depicting the distribution of pore sizes of AC, MF, MF25-AC, MF45-AC, MF65-AC and Pure MF.

The porosity including pore size and surface area of as-synthesized materials were measured at a temperature of –196 °C through N_2_ sorption isotherms as a standard tool for characterization and displayed in Fig. 3. The isotherms for pure and modified activated carbon using several weight percentages of magnesium ferrites have a Type I isotherm, which reveals that pure and modified AC were essentially microporous according to the IUPAC classification. As well as, the figure showed that most of pore volumes of AC, MF25-AC, MF45-AC and MF65-AC were filled below P/P^o^ equal 0.1, proving that the prepared samples were highly microporous. The specific surface area (S_BET_) of pure AC derived from orange peels reached 2134 m^2^ g^–1^, while pure MF has a surface area of 178 m^2^ g ^–1^, which is well fitted with the previously published literatures^32,71^. After modification with magnesium ferrites, the S_BET_ (Table 1) was reduced from 1897 to 1645 to 1223 m^2^.g^–1^ for MF25-AC, MF45-AC and MF65-AC, respectively. The noted reduction in specific S_BET_ can be ascribed to the accumulation of MF-NPs on surface and within the pores of the activated carbon (AC). This deposition, in turn, leads to a decrease in nitrogen adsorption and a consequent decline in the overall surface area^35^. Moreover, the pore size distribution curves of AC, MF25-AC, MF45-AC and MF65-AC was studied using the pore volumes in the measurement of N_2_ desorption isotherms^72^. All the prepared models have a narrow pore size distribution with pore diameter less than 20 Å as displayed in Fig. 4, that confirm the micropores existed in the pure and modified activated carbons. The average pore diameter (Dp) (Table 1) decreased with increasing the contents of magnesium ferrites which was found to be 18.7, 6.8, 17.4, 16.1 and 13.5 Å for AC, MF, MF25-AC, MF45-AC and MF65-AC, respectively. This decrease can be due to the deposition of MF NPs in the pores of activated carbons^63^.

### TEM Images and particle distribution histogram

**Figure 5:**
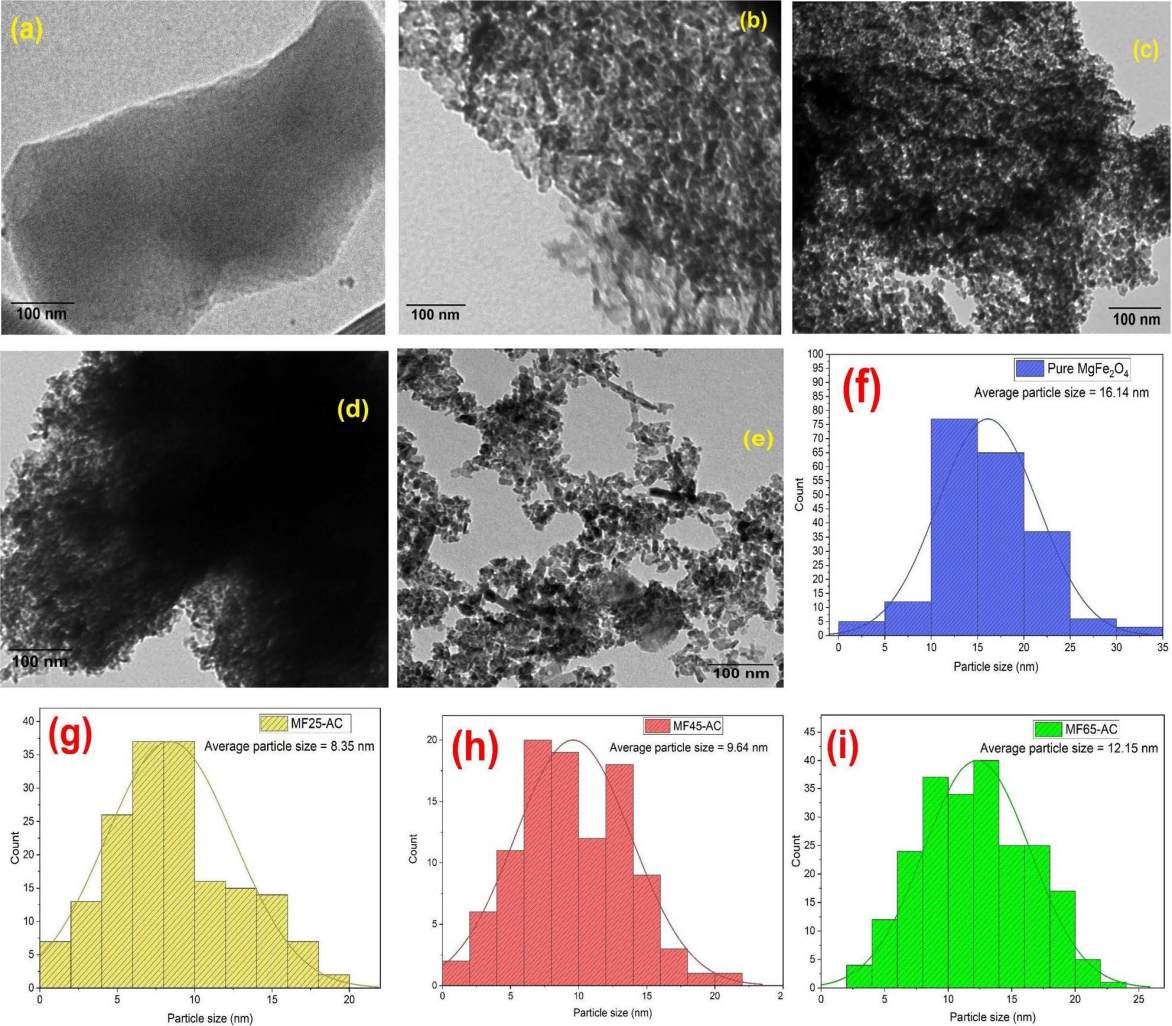
TEM images of (**a**) pure AC, (**b**) pure MF, (**c**) MF25-AC, (**d**) MF45-AC and (**e**) MF65-AC; (**f**–**i**) particle size histogram of MF, MF25-AC, MF45-AC and MF65-AC.

Figure 5 displayed the TEM images of AC, MF, MF25-AC, MF45-AC and MF65-AC. According to the TEM images, AC was appeared as a sheet like structure with obvious micropores observed on the surface of it (Fig. 5a). While, MgFe_2_O_4_ nanoparticles in Fig. 5b has a spherical nanoparticle like composition with a particle size ranged from 12.4 to 17.7 nm and there are some rods were detected in the TEM images. After adding magnesium ferrites to AC (Fig. 5c–e), the spherical and rods nanoparticles were found to be well dispersed on the surface of AC and with increase the contents of MF, the particles were accumulated on the surface that could block the pores of AC as shown in MF65-AC sample and these results were fitted with BET analysis. On the other hands, image J software was used to investigate the average nanoparticle size of MF in pure MF and other supported MF samples were and exhibited in Fig. 5f–i. The average particle size histograms displayed that the average particle sizes of MgFe_2_O_4_ NPs (Table 1) were equaled to be 16.14, 8.35, 9.64, and 12.1 nm for pure MF, MF25-AC, MF45-AC, and MF65-AC, respectively. These findings were well matched with XRD results.

### SEM images, energy dispersive spectroscopy (EDX) and EDX-mapping analysis

**Figure 6:**
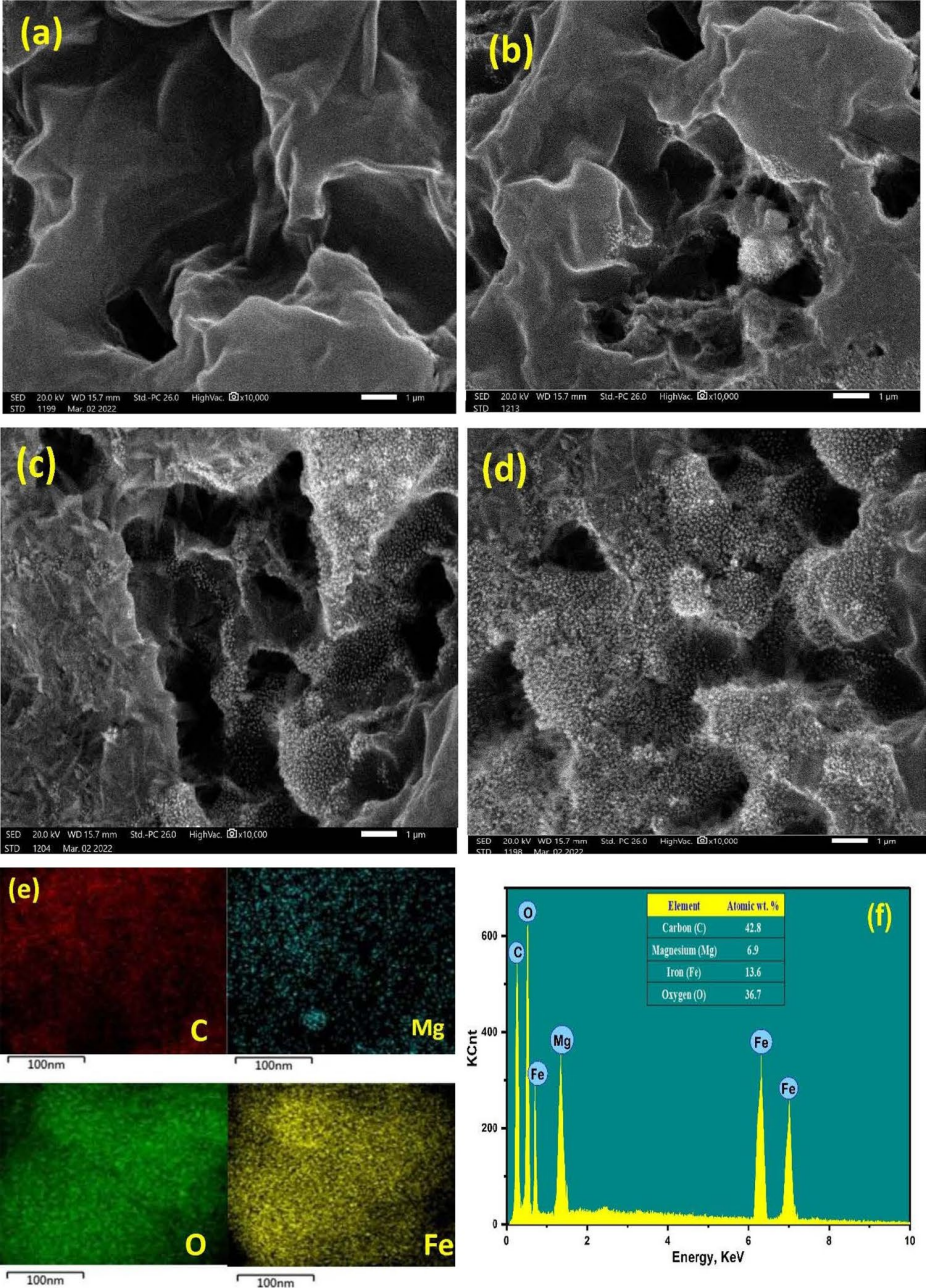
(**a**–**d**) SEM images of AC, MF25-AC, MF45-AC and MF65-AC; (**e**) EDX-Maps of MF45-AC; and (**f**) EDX spectrum of MF45-AC.

The surface morphology was studied using SEM images, while EDX analysis was applied to study their chemical composition. Pure activated carbon (Fig. 6a) has a layer like structure with high porosity as was previously reported^30^. While, other supported AC displayed highly distributed spherical nanoparticles on the surface of AC which were further enlarged in size upon increasing their percentage content as were shown in Fig. 6b–d. These reveals that MgFe_2_O_4_ NPs were successfully homogenously supported on ACs with high dispersion characteristics as was supported from the EDX mapping. Figure 6e displayed that magnesium and iron were randomly distributed in structure of the prepared composites. This good dispersion could responsible for the excellent electrochemical performance that will be displayed later. Also, EDX analysis was displayed in Fig. 6f, which showed that only four peaks were observed in MF45-AC spectrum that were attributed to carbon, oxygen, magnesium and iron. These results reveal that MF45-AC was successfully prepared without contaminations.

### Electrochemical performance

**Figure 7:**
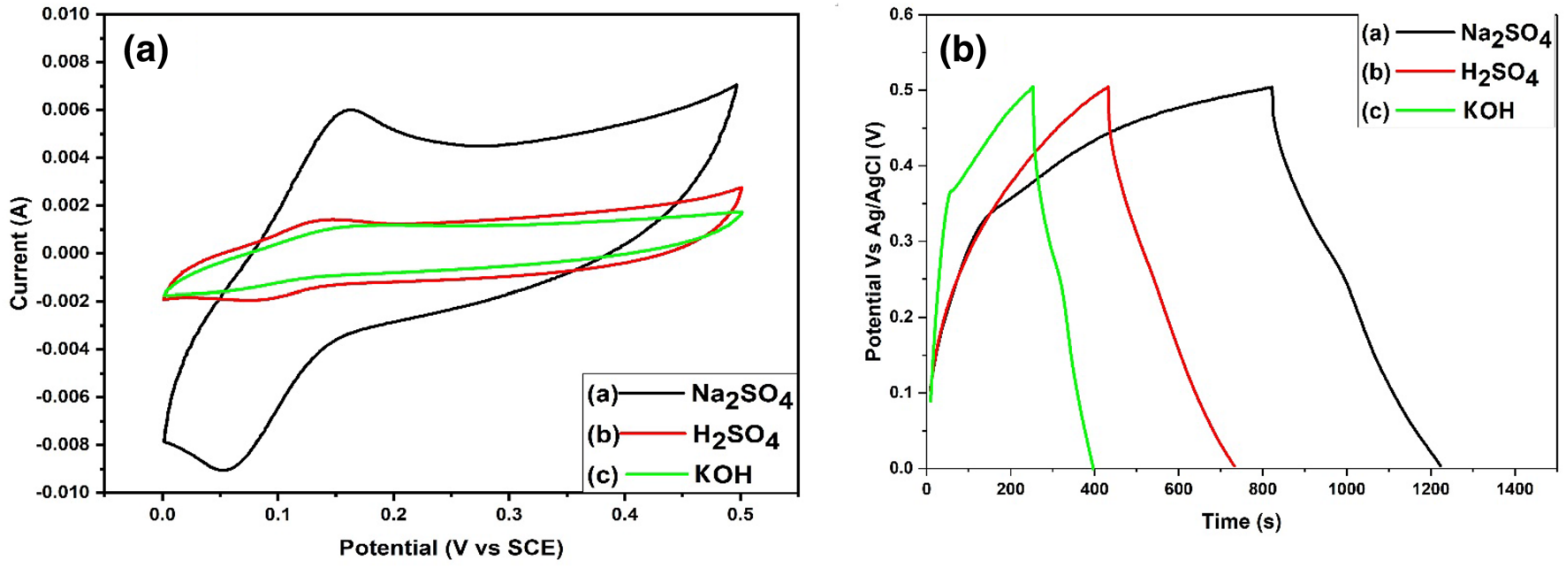
(a) cyclic voltammetry (CV) and (b) galvanostatic charge–discharge (GCD) curves of MF45-AC in various electrolyte solutions (KOH, H_2_SO_4_, and Na_2_SO_4_).

The electrolyte used for conducting cyclic voltammetry (CV) and galvanic charge–discharge measurements (GCD) encompassed three different media: a 2.0 M solution of sodium sulfate (Na_2_SO_4_) for neutral conditions, sulfuric acid (H_2_SO_4_, 2.0 M) for acidic conditions, and potassium hydroxide (KOH, 2.0 M) for alkaline conditions. These electrolytes were selected based on their compatibility with the materials under investigation and their relevance to supercapacitor research, allowing for a comprehensive evaluation of the electrochemical performance across different pH environments. The electrochemical performances (CV) were examined at a scan rate of 20 mV/sec over the MF45-AC composite using these electrolytes, and the results are depicted in Fig. 7a. Comparing Na_2_SO_4_ solution to the other solutions, a greater electrochemical active region was discovered. Therefore, Na_2_SO_4_ was chosen as the most appropriate electrolyte. As well as, GCD behavior of MF45-AC nanocomposite using different electrolytes (KOH, H_2_SO_4_, and Na_2_SO_4_) was investigated through galvanostatic charging and discharging (GCD) tests (Fig. 7b). demonstrated distorted triangular or quasi-triangular curves, indicative of the redox behavior of magnesium ferrite nanoparticles supported on activated carbon. The specific capacitance of MF45-AC was calculated from Eq. (2) and was found to be 292, 598 and 870 F g^-1^ at a current density of 1.0 A g^-1^ in KOH, H_2_SO_4_, and Na_2_SO_4_ solutions, respectively. The results of GCD curves were fitted with the CV data confirming that Na_2_SO_4_ (2.0 M) was the most suitable electrolyte in electrochemical measurements.

**Figure 8:**
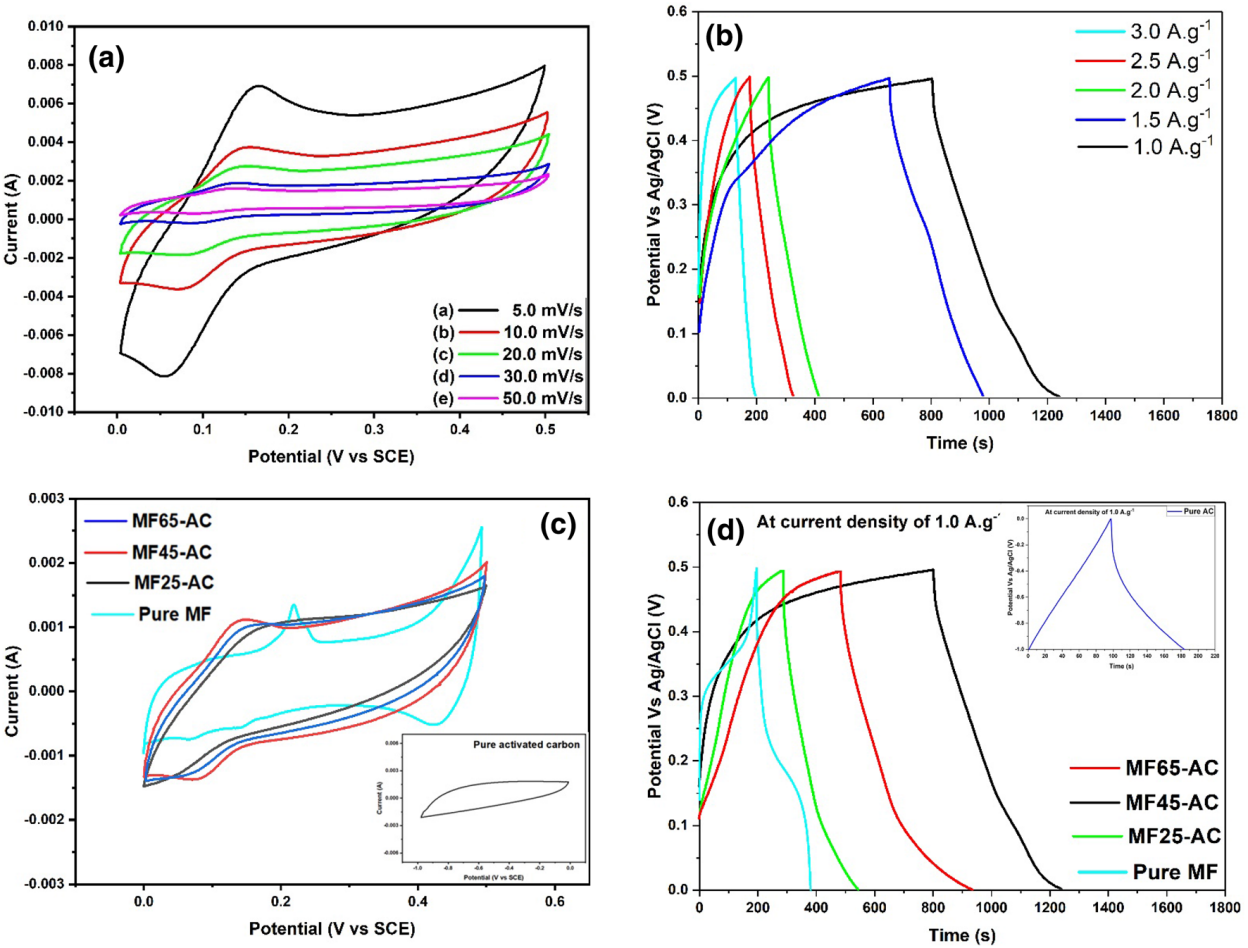
(**a**) CV curves at different scan rates and (**b**) GCD curves at different current density of MF45-AC, (**c**) CV curves of AC, MF, MF25-AC, MF45-AC, and MF65-AC at a scan rate of 20.0 mV/s, and (**d**) GCD curves of all prepared electrode at 1.0 A. g^-1^.

The cyclic voltammetry measurements of MF45-AC electrode were tested in a 2.0 M Na_2_SO_4_ electrolyte concentration at various scan rates ranging from 5.0 to 50.0 mV s^-1^ as shown in Fig. 8a. The figure displayed that there is an increase in current intensity with scanning rate, indicating good rate capability. Also, MF45-AC showed exceptional redox peaks at 0.091 and 0.238 V, that could responsible for the excellent electrochemical performance. This observation indicates the presence of significant Faradaic redox reaction effects, suggesting a considerable influence on the capacitance and pseudocapacitive properties of the electrode material. The Faradaic reactions contribute to the overall electrochemical behavior, showcasing the involvement of redox processes that can enhance the capacitive performance and pseudocapacitive characteristics of the MF45-AC composite. Alternatively, Fig. 8b displayed the GCD curves of MF45-AC at different current density ranged from 1.0 to 3.0 A g^-1^ at the same potential windows. The quasi-triangular shape evident in all galvanostatic charge–discharge (GCD) curves further confirms the redox behavior of the prepared materials, consistent with the observations in cyclic voltammetry (CV) curves. The specific capacitance of MF45-AC was found to be 870, 790, 668, 585, and 396 F·g^−1^ at current densities of 1.0, 1.5, 2.0, 2.5, and 3.0 A·g^−1^, respectively. This characterization provides insights into the dynamic response and energy storage capabilities of the MF45-AC composite under various operational conditions. As well as, Fig. 8c illustrates the cyclic voltammetry (CV) curves of AC, MF, MF25-AC, MF45-AC, and MF65-AC at a scan rate of 20.0 mV/s. The CV curves of AC exhibit rectangular shapes within a potential window of − 1.0 to 0 V, indicating their typical double electric layer behavior and ideal capacitive characteristics. In contrast, the CV curves of the other ferrite composites, namely MF, MF25-AC, MF45-AC, and MF65-AC, reveal faradaic-type capacitive behavior. This behavior arises from the oxidation or reduction processes of active materials occurring at the electrode interface, indicating the involvement of redox reactions in the electrochemical performance of these composite materials as shown in Fig. 8d. AC demonstrates triangular-shaped GCD curves, which are characteristic of double electric layer behavior and ideal capacitive properties as confirmed by the CV curves. On the contrary, the ferrite samples display quasi-triangular-shaped galvanostatic charge–discharge (GCD) curves, a characteristic that signifies their redox behavior. This observation suggests the involvement of redox reactions in the charge storage mechanism of the ferrite samples, distinguishing them from the ideal capacitive behavior typically associated with double-layer capacitors. The specific capacitance was calculated using Eq. (2) and found to be 96 F·g^-1^ for AC, 402 F·g^-1^ for MF, 516 F·g^-1^ for MF25-AC, 870 F·g^-1^ for MF45-AC, and 800 F·g^-1^ for MF65-AC at a current density of 1.0 A·g-1. The specific capacitance increases within the composites and continues to rise with increasing magnesium ferrite content until reaching a maximum value at 45.0 wt.%. After this point, the specific capacitance starts to decrease again. This decrease can be attributed to the accumulation of ferrite nanoparticles on the surface of activated carbon, which obstructs the porous structure of AC and hinders the diffusion of ions from the electrolyte within the electrode materials.

**Figure 9:**
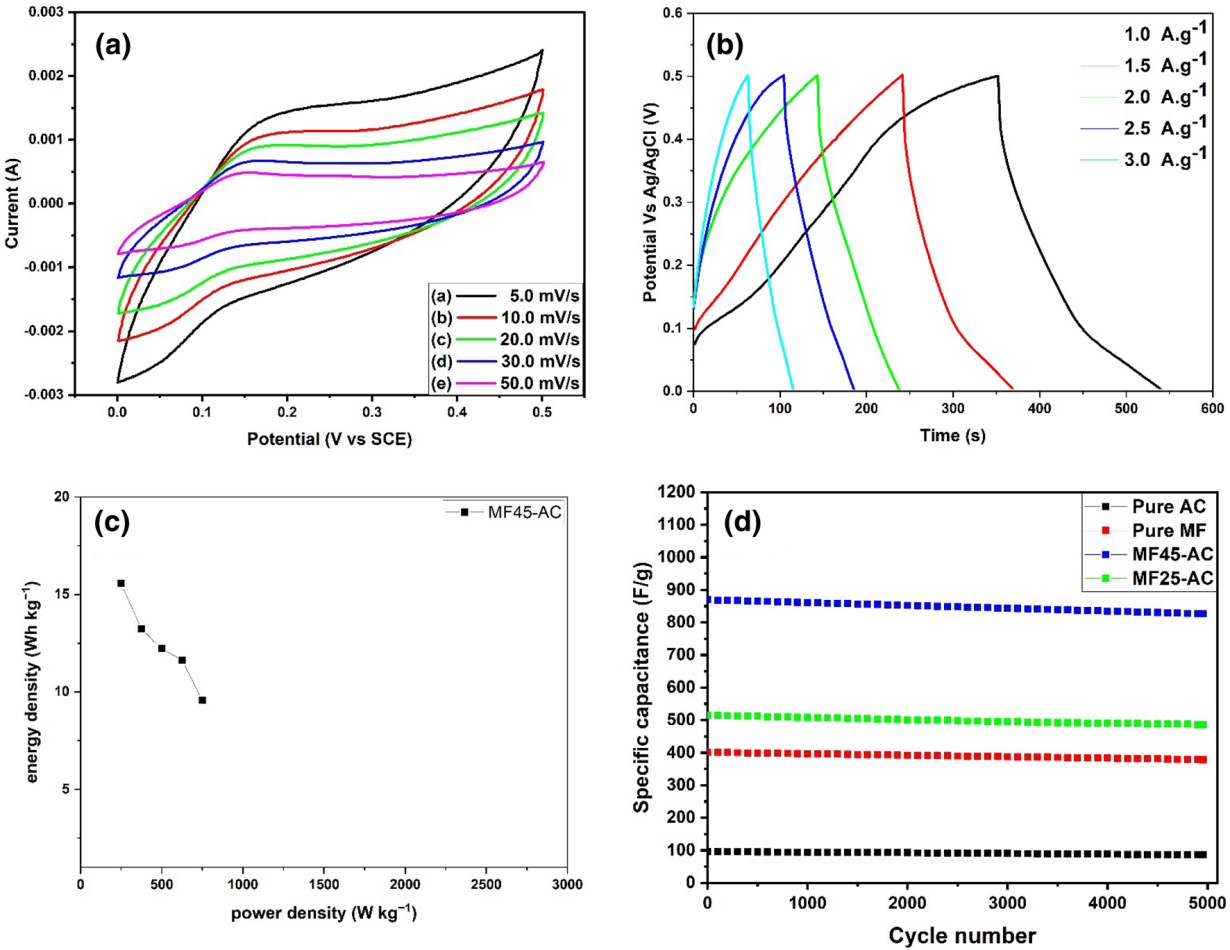
(**a**) CV curves at various scan rates, (**b**) GCD curves at various current density, (**c**) Ragone plot of ASC MF45-ACelectrode, and (**d**) cyclic stability of AC, MF, MF25-AC, and MF45-AC electrodes.

To evaluate the electrode's practical performance, CV analysis of the asymmetric MF45-AC electrode was conducted within a potential window spanning from 0.0 to 0.5 V, employing various scan rates from 5.0 to 50.0 mV/s, as depicted in Fig. 9a. The shape of the CV curves for MF45-AC appears distorted and rectangular, indicating significant contributions from Faradaic redox reactions and pseudocapacitive properties. Additionally, the GCD curves of the asymmetric MF45-AC electrode were acquired under various current densities (1.0 to 3.0 A·g^-1^) and displayed in Fig. 9b. This analysis was crucial in assessing the electrode's performance and energy storage capabilities at different operational conditions. The obtained GCD curves offer valuable information about the charge and discharge behavior, as well as the stability and efficiency of the asymmetric MF45-AC electrode under diverse current density regimes. The specific capacitances of MF45-AC were calculated at 1.0, 1.5, 2.0, 2.5, and 3.0 A·g^-1^ using Eq. (2) and found to be 448, 381, 352, 325, and 276 F·g^-1^, respectively. The Ragone plot, depicting the relationship between energy density and power density, was generated for the asymmetric MF45-AC electrode based on the galvanostatic charge–discharge (GCD) curves obtained at different current densities, as illustrated in Fig. 9c. The maximum energy density of MF45-AC was identified at 15.56 W·h·kg^−1^ with a corresponding power density of 250 W·kg^−1^. This energy density value proves to be comparable to or even superior to those reported for other asymmetric devices, underscoring the favorable performance of the MF45-AC electrode in balancing energy storage and delivery in practical applications^73–75^. As well as, cycle stabilities of the pure AC, pure MgFe_2_O_4_, and MF45-AC electrodes at current density equal 3.0 A g^−1^ is presented in Fig. 9d. According to Fig. 9d, all the prepared samples showed an increase in the electrochemical performance in the first 500 cycle, which could be due to the activation process of the electrode. After 5000 cycles, the performance of the electrodes displays a decrease in specific capacitance until reached to 86.1, 378.4, and 826.5 F. g^–1^ in case of pure AC, pure MgFe_2_O_4_, and MF45-AC electrodes, respectively. According to these results, the capacitance retention of pure AC, pure MgFe_2_O_4_, and MF45-AC electrodes was founded to be 89.6%, 94.2%, and 95.1%, respectively. These results suggested that the prepared electrodes displayed excellent cycle stability even after 5000 cycles and MF45-AC has the highest electrochemical performance and cycle stability. The electrochemical performance of our recent study (MgFe_2_O_4_/activated carbon nanocomposites electrodes) is compared with others related supercapacitor electrodes published in the previous literatures and illustrated in Table 2. The results show that MF45-AC nanocomposite act as effective and promising supercapacitors in terms of electrochemical performance.

**Table 2:** electrochemical performance of different supercapacitor electrode materials based on activated carbon and metals oxides supported on different supports reported in previous works.

Active material	Electrolyte	Substrate	Specific capacitance	Capacitance retention	[Ref]
Activated carbon derived from orange peel	1.0 m H_2_SO_4_	–	460.0 f g^-1^	98%	^76^
Activated carbon	0.1 m H_2_SO_4_	Glassy carbon	275.0 f g^-1^	–	^77^
Magnesium ferrite	3.0 m KOH	Nickel foam	250.0 f g^-1^	–	^78^
Magnesium ferrite	–	–	190.0 f g^-1^	–	^79^
MgO_2_/activated carbon	1.0 m H_2_SO_4_	–	579.7 f g^-1^	–	^80^
MgO_2_/activated carbon paper	1.0 m Na_2_SO_4_	–	485.4 f g^-1^	85%	^81^
ZnFe_2_O_4_/activated carbon	3.0 m KOH	Nickel foam	609.0 f g^-1^	91%	^82^
MnFe_2_O_4_/activated carbon	PVA/KOH gel	Nickel foam	420.0 f g^-1^	98%	^83^
MgFe_2_O_4_/activated carbon	2.0 Na_2_SO_4_	Graphite sheet	870.0 f g^-1^	95.1%	our work

## Conclusion

Activated carbon was successfully prepared from orange peels as agricultural wastes, then modified with different weight contents of magnesium ferrites (25, 45 and 65 wt.%) through simple solvothermal technique. The as-synthesized composites were characterized by different characterization techniques, in which the surface area of AC was calculated from nitrogen adsorption isotherms and founded to be 2134 m^2^ g^–1^ and decreased with increasing the ferrites contents until reached to 1423 m^2^ g^–1^ at MF65-AC. Additionally, SEM and TEM images prove that the ferrites nanoparticles was exceptionally well dispersed on the surface of AC. The electrodes synthesized in their as-prepared state exhibited significant enhancements in electrochemical performance, demonstrating a notable improvement as the magnesium ferrite content increased in the MF45-AC composite. This observation underscores the positive impact of incorporating magnesium ferrites on the overall electrochemical properties of the composite electrode. This optimum compositional content showed highest C_s_ with value of 870.0 F g^-1^ at current density of 1.0 A, and exceptional retention with 95.1% of its initial specific capacitance even after 5000 cycles. Also, it showed asymmetric specific capacitance equal to 448 F g^–1^ at current density of 1.0 A g^-1^ and the energy of this asymmetric electrode was reached to 15.56 W h kg^-1^ at power density 250 W kg^-1^. Increasing the content beyond the 45 wt.% up to 65 wt. % demolished the electrochemical performance due to excessive clogging of the pores of AC by ferrites nanoparticles as confirmed by TEM images as well as the pronounced decrease in the surface area. Our future research endeavors will focus on exploring alternative synthesis routes and modifying the composite formulations. Additionally, we aim to investigate the mechanistic aspects of the redox reactions, understanding the dynamics that contribute to the exceptional capacitance observed. By systematically investigating and optimizing these parameters, we anticipate advancing the design of magnesium ferrite-activated carbon composites for enhanced energy storage applications.

## Data Availability

The datasets used and/or analysed during the current study are available from the corresponding author upon request.
