# Author Correction: Synthesis and characterization of magnesium ferrite-activated carbon composites derived from orange peels for enhanced supercapacitor performance

**DOI:** 10.1038/s41598-024-60087-6

**Published:** 2024-04-30

**Authors:** Reda. S. Salama, Mostafa S. Gouda, Mohamed F. Aly Aboud, Fares T. Alshorifi, A. A. El‑Hallag, Ahmad K. Badawi

**Affiliations:** 1 Basic Science Department, Faculty of Engineering, Delta University for Science and Technology, Gamasa, Egypt; 2 Department of Mechanical Engineering, Faculty of Engineering, Islamic University of Madinah, P.O.B. 170, 42351 Madinah, Saudi Arabia; 3 Department of Chemistry, Faculty of Science, University of Saba Region, Marib, Yemen; 4 Department of Chemistry, Faculty of Science, Sana’a University, Sana’a, Yemen; 5 Civil Engineering Department, El-Madina Higher Institute for Engineering and Technology, Giza, 12588 Egypt

Correction to: *Scientific Reports* 10.1038/s41598-024-54942-9, published online 08 April 2024

In the original version of this article author A. A. El-Hallag, was incorrectly affiliated with ‘Department of Mechanical Engineering, Faculty of Engineering, Islamic University of Madinah, P.O.B. 170, 42351, Madinah, Saudi Arabia’.

The correct affiliation is listed below:

Basic Science Department, Faculty of Engineering, Delta University for Science and Technology, Gamasa, Egypt

The original Article has been corrected.

